# Stage-specific Effects of Bioactive Lipids on Human iPSC Cardiac Differentiation and Cardiomyocyte Proliferation

**DOI:** 10.1038/s41598-018-24954-3

**Published:** 2018-04-26

**Authors:** Arun Sharma, Yuan Zhang, Jan W. Buikema, Vahid Serpooshan, Orlando Chirikian, Nina Kosaric, Jared M. Churko, Elda Dzilic, Alice Shieh, Paul W. Burridge, Joseph C. Wu, Sean M. Wu

**Affiliations:** 1 Stanford Cardiovascular Institute, Stanford University School of Medicine, Stanford CA, 94305 USA; 2 Institute for Stem Cell Biology and Regenerative Medicine, Stanford University School of Medicine, Stanford CA, 94305 USA; 3 Department of Cardiology, University Medical Center Utrecht, Utrecht University, 3508 GA Utrecht, The Netherlands; 4 Department of Cardiovascular Surgery, German Heart Centre Munich, Technical University Munich, Munich, Germany; 5 Department of Medicine, Division of Cardiology, Stanford University School of Medicine, Stanford CA, 94305 USA; 6 Present Address: Department of Genetics, Harvard Medical School, Boston, MA 02115 USA; 7 Present Address: Department of Pharmacology and Center for Pharmacogenomics, Northwestern University Feinberg School of Medicine, Chicago, IL USA

## Abstract

Bioactive lipids such as sphingosine-1-phosphate (S1P) and lysophosphatidic acid (LPA) regulate diverse processes including cell proliferation, differentiation, and migration. However, their roles in cardiac differentiation and cardiomyocyte proliferation have not been explored. Using a 96-well differentiation platform for generating human induced pluripotent stem cell-derived cardiomyocytes (hiPSC-CMs) we found that S1P and LPA can independently enhance cardiomyocyte generation when administered at an early stage of differentiation. We showed that the combined S1P and LPA treatment of undifferentiated hiPSCs resulted in increased nuclear accumulation of β-catenin, the canonical Wnt signaling pathway mediator, and synergized with CHIR99021, a glycogen synthase kinase 3 beta inhibitor, to enhance mesodermal induction and subsequent cardiac differentiation. At later stages of cardiac differentiation, the addition of S1P and LPA resulted in cell cycle initiation in hiPSC-CMs, an effect mediated through increased ERK signaling. Although the addition of S1P and LPA alone was insufficient to induce cell division, it was able to enhance β-catenin-mediated hiPSC-CM proliferation. In summary, we demonstrated a developmental stage-specific effect of bioactive lipids to enhance hiPSC-CM differentiation and proliferation via modulating the effect of canonical Wnt/β-catenin and ERK signaling. These findings may improve hiPSC-CM generation for cardiac disease modeling, precision medicine, and regenerative therapies.

## Introduction

Adult mammalian cardiomyocytes possess limited capacity for cell division^1^. Radiocarbon dating studies suggest that there is, at baseline, less than 0.5% yearly cell turnover in the adult human heart^2^. As such, mammalian adult heart regeneration is unable to compensate for the massive loss of cardiomyocytes following cardiac injury such as myocardial infarction, leading to adverse cardiac remodeling. This limited regenerative capability of the human heart has garnered significant interest in developing novel methodologies for both creating cardiomyocytes *de novo* and inducing proliferation in terminally differentiated cardiomyocytes. A major goal in human pluripotent stem cell research is to provide large quantities of cardiomyocytes suitable for cellular therapy in regenerative medicine^3–6^.

Protocols for human pluripotent stem cell cardiac differentiation are vastly improved compared to a decade ago. Current protocols can obtain upwards of 90% pure cardiomyocytes during differentiation followed by metabolic selection, which can be further augmented by using CRISPR/Cas9 gene editing to introduce selectable markers into hiPSCs^7–9^. The most up-to-date strategies use biphasic Wnt/β-catenin modulation for direct cardiac differentiation from human induced pluripotent stem cells (hiPSCs)^10,11^. To mimic developmental Wnt signals required for *in vivo* mesoderm induction, hiPSCs are initially treated with CHIR99021 (CHIR), a non-selective glycogen kinase 3 beta (GSK3β) inhibitor, followed by a Wnt/β-catenin inhibitor to promote cardiac cell differentiation. However, despite recent advances, there remains significant batch-to-batch variation in differentiation efficiency, as different hiPSCs lines, even those derived from the same individuals, can vary in their abilities to reproducibly generate cardiomyocytes. To address the challenge of *in vitro* hiPSC-CM generation with consistently high efficiency, we sought novel molecules that can further optimize our current cardiac differentiation protocol.

In recent years, growing evidence support lysophospholipids, a collection of bioactive lipids harboring multiple functions, as important regulators of stem cell differentiation *in vitro* and cardiovascular development *in vivo*^12^. Among these bioactive lipids, sphingosine-1-phosphate (S1P) and lysophosphatidic acid (LPA) are cardinal members. *In vivo* studies have demonstrated a necessary role of S1P signaling via S1P receptor in cardiomyocytes in normal heart development in mice^13^. *In vitro* studies have shown that these signaling molecules are capable of regulating pluripotency and cell cycle activity in human embryonic stem cells^14–16^. The bioactive lipids have also been reported to play a role in cell proliferation in epithelial cells, fibroblasts, and various cancer cell lines, via their ability to stimulate important cellular signaling pathways such as the MAPK/ERK pathway, the Hippo Pathway, and the Wnt/β-catenin signaling pathway^17–20^. These diverse roles for bioactive lipids prompted us to consider whether S1P and LPA may regulate cardiomyocyte differentiation from hiPSCs and cardiomyocyte function.

In this study, we explored S1P and LPA as inducers of cardiomyocyte differentiation in a chemically-defined culture setting using multiple hiPSC lines. Furthermore, we examined lysophospholipid regulation of cardiomyocyte progeny from hiPSCs and their ability to stimulate cardiomyocyte proliferation. We found that S1P and LPA act synergistically with GSK3β inhibitor CHIR to regulate early hiPSC mesodermal differentiation through nuclear β-catenin accumulation. At later stages, the combined treatment of S1P and LPA resulted in cell-cycle activation in differentiated hiPSC-CMs, an effect mediated through ERK/MAPK signaling, and synergized with β-catenin signaling to increase cardiomyocyte proliferation. Taken together, our findings demonstrate unequivocally that bioactive lipids exhibit stage-specific effects on cardiac differentiation from hiPSCs.

## Results

### Bioactive lipids augment cardiac differentiation from hiPSCs in a stage-specific manner

Five hiPSC lines were generated through reprogramming somatic tissues from five individuals by introducing viral vectors expressing the Yamanaka reprogramming factors (*OCT4*, *SOX2*, *KLF4*, and c-*MYC)*. Subsequently, all hiPSC lines were differentiated using chemically-defined protocols to generate cardiomyocytes^10,21^. Since hiPSC lines 3, 4, and 5 differentiated well into beating cardiomyocytes without the addition of bioactive lipids, we investigated the effects of S1P/LPA on hiPSC lines 1 and 2 that exhibited impaired capacity to differentiate into cardiomyocytes.

**Figure 1:**
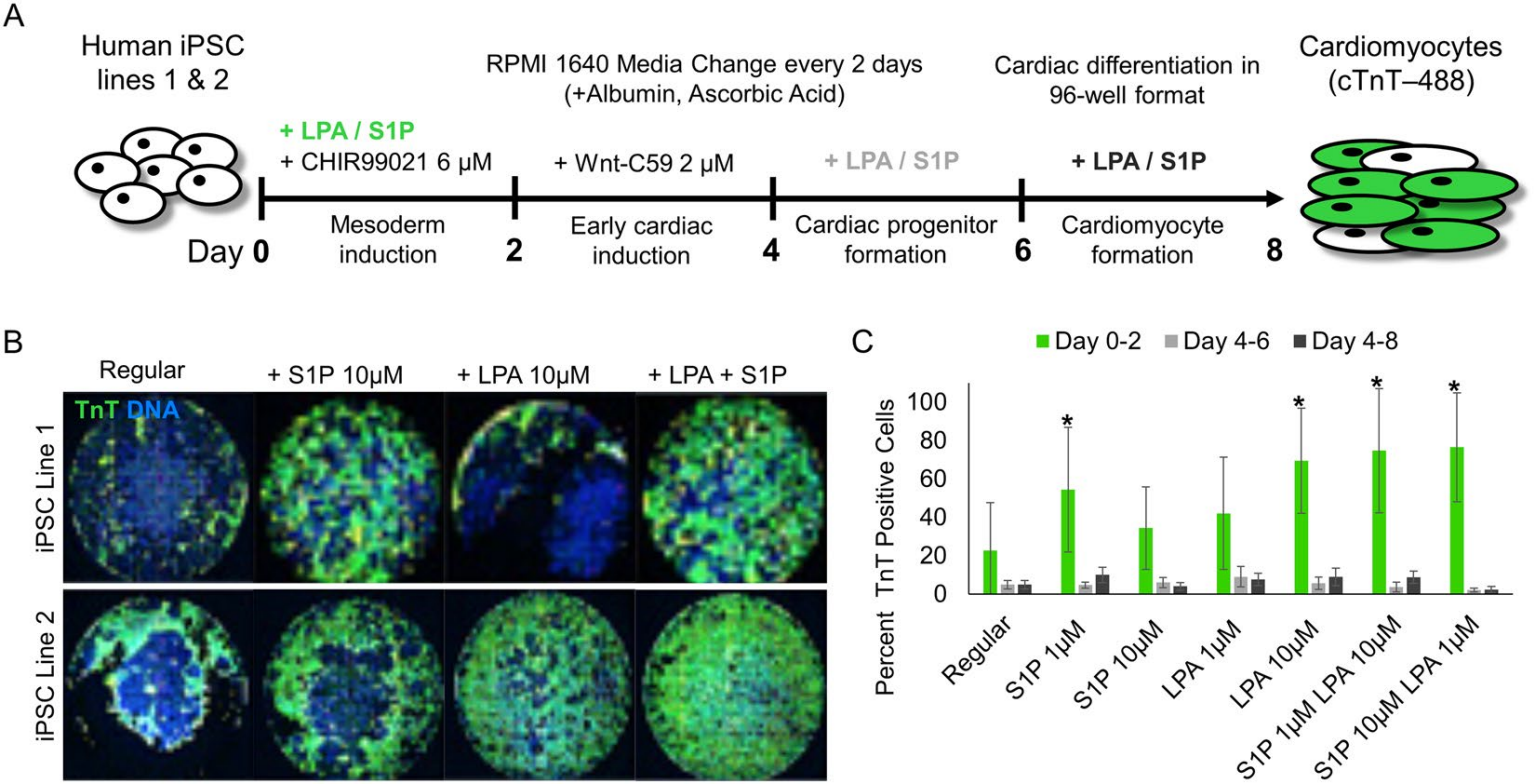
96-well differentiation illustrates S1P/LPA-mediated enhancement of hiPSC-cardiomyocyte differentiation when added concurrently with Wnt activator CHIR99021. (**A**) Illustration of the ‘regular’ chemically-defined cardiac differentiation protocol utilized in this study. S1P/LPA was added at different time points during hiPSC-CM differentiation. (**B**) Representative 96-well immunofluorescence images for cardiac troponin T (TnT) in green and nuclear DNA in blue of 2D monolayer-based, chemically-defined differentiation of two poorly differentiating hiPSC lines into cardiomyocytes. Staining was performed in a 96-well plate format on day 8-post differentiation hiPSC-CMs. S1P, LPA, or both were added for days 0–2, 4–6 or 6–8 during the hiPSC-CM differentiation process. (**C**) Quantification of TnT positive cell numbers of total represented as percentages TnT positive cells for each time point when S1P, LPA or both were added. Error bars represent standard deviation. * indicates p < 0.05 versus control. Experiments were performed in 2 different hiPSC lines in 3–6 replicates.

We first examined whether S1P and LPA treatment could improve cardiomyocyte differentiation in these two hard-to-differentiate hiPSC lines. We established a 96-well differentiation platform to assess the efficiency of CM differentiation upon treatment with bioactive lipids (Fig. 1A). Using this platform, we determined that the addition of S1P and/or LPA concurrently with CHIR between days 0–2 in the chemically-defined differentiation protocol enhanced hiPSC-CM generation by 2–3 fold in comparison to control, as assessed by cardiac troponin T (TnT) expression at day 8^10,21^ (Fig. 1B,C). We did not see significant enhancement of hiPSC-CM differentiation when S1P/LPA were added between days 4–6 or 6–8 of differentiation (Fig. 1C). These results indicate that bioactive lipids have an early role in augmenting cardiac differentiation and cardiomyocyte generation in these otherwise poorly differentiating hiPSC lines.

### Bioactive lipids synergize with Wnt/β-catenin in hiPSCs to induce mesodermal differentiation

**Figure 2:**
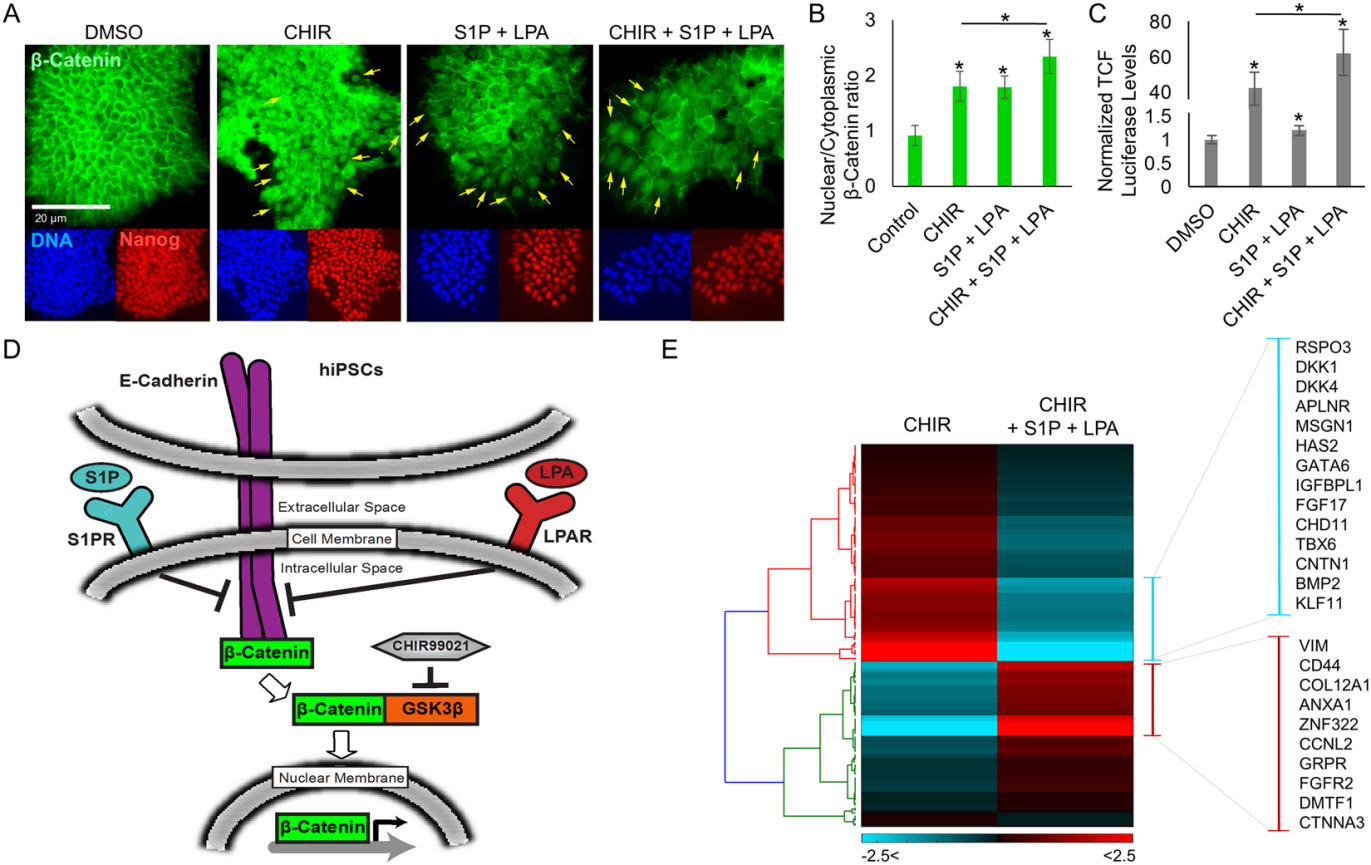
Bioactive lipids S1P and LPA enhance β-catenin nuclear accumulation and activate Wnt signaling during early cardiac differentiation from hiPSCs. (**A**) Immunofluorescence for β-catenin (green), pluripotency marker Nanog (red), and DAPI (DNA) (blue) following 2-hour treatment of hiPSCs with DMSO, small molecule GSK3β inhibitor/Wnt activator CHIR99021 (CHIR), bioactive lipids S1P + LPA, or CHIR + bioactive lipids. Arrows indicate cells exhibiting characteristic β-catenin nuclear accumulation. (**B**) Quantification of β-catenin staining represented as nuclear intensity over cytoplasmic intensity for the treatment groups normalized to DMSO control. (**C**) Luciferase luminescence intensity after transfection of hiPSCs with TOPFlash Wnt pathway activity reporter and 2-hour treatment with CHIR, S1P/LPA, or both, represented as fold increase over DMSO control. (**D**) Model illustrating the signaling cascade linking bioactive lipids and the Wnt/β-catenin signaling pathway in the context of hiPSCs. Treatment with S1P/LPA on hiPSCs dissociates β-catenin from adherens junctions and E-cadherin, thus increasing the overall β-catenin pool that can be utilized for downstream signaling and gene transcription. Treatment with GSK3β inhibitor CHIR frees β-catenin and increases the overall intracellular β-catenin pool for downstream signaling and gene transcription. (**E**) Microarray analysis illustrating key alterations in gene expression following 48-hour treatment of hiPSCs with small molecule GSK3β inhibitor/Wnt activator CHIR with or without bioactive lipids S1P/LPA. A list of up- (red) and down- (blue) regulated genes after treatment with bioactive lipid is shown. Experiments were performed in 3–4 biological replicates.

To generalize our findings beyond these two poorly differentiating hiPSC lines, we focused the remainder of our studies on our three independent hiPSC lines that exhibited normal capacity of cardiac differentiation. The treatment of undifferentiated hiPSCs with inducers of Wnt/β-catenin signaling has previously been shown to effectively enhance mesodermal differentiation^10^. Since we found that hiPSC-CM differentiation is enhanced when bioactive lipids were administered early, we hypothesized that the observed augmentation of hiPSC-CM formation could be attributed to an increase in the level of nuclear β-catenin by S1P/LPA. Previous work in other cell lines showed that bioactive lipids enhance β-catenin dissociation from E-cadherin at adherens junctions, increasing the cytoplasmic β-catenin available for downstream signaling in the nucleus^22^. Given this, we examined whether S1P/LPA treatment of undifferentiated hiPSCs, either alone or in combination with CHIR, could increase the level of nuclear β-catenin. First, we noted that S1P/LPA treatment alone induced significant nuclear accumulation of β-catenin in hiPSCs (Fig. 2A,B). Additionally, serial immunostaining for β-catenin at different time points revealed that this effect could be observed as early as within 2 hours after treatment (Fig S1A). When combined with CHIR, S1P and LPA further promoted β-catenin nuclear accumulation, suggesting a synergy between these compounds (Fig. 2A,B). To examine whether the increase in nuclear β-catenin level results in an activation of Wnt signaling at the transcriptional level, we transfected hiPSCs with the previously described TOPFlash luciferase reporter plasmid^23^. This system provides a proportionate visualization of Wnt transcriptional activity using bioluminescence. Subsequently, we treated the cells with dimethyl sulfoxide (DMSO), CHIR, S1P/LPA, or a combination of S1P/LPA and CHIR. Interestingly, when compared to the DMSO control group, treatment with S1P/LPA alone minimally increased TCF/LEF-luciferase activity (~1.25-fold) (Fig. 2C) whereas treatment with CHIR alone led to a highly significant ~40-fold increase in TCF/LEF-luciferase activity. When S1P/LPA was combined with CHIR, the TCF/LEF-luciferase activity was further increased to more than 60-fold compared with DMSO control (Fig. 2C). Hence, while S1P/LPA treatment alone minimally activated the LEF/TCF reporter, the bioactive lipids synergized with CHIR to further increase β-catenin signaling (Fig. 2D)

To uncover the key changes in gene expression after S1P/LPA treatment, we performed a genome-wide microarray expression analysis on hiPSCs at day 2 of differentiation. Interestingly, we found a significant downregulation of *DKK4* and *DKK1* (Fig. 2E), Wnt inhibitors that are well known to regulate stem cell development and differentiation^24^. In addition, our microarray data also uncovered an increase in the expression of cytoskeletal and extracellular matrix genes such as COL12A1 and Vimentin (*VIM*), the latter of which is associated with an epithelial-to-mesenchymal transition during development.

Taken together, these results suggest that bioactive lipids S1P and LPA act synergistically with GSK3β inhibitor CHIR to increase Wnt signaling/β-catenin nuclear accumulation during hiPSC differentiation, potentially through enhancing β-catenin release from membrane-associated E-cadherin (Fig. 2D). S1P and LPA treatment also suppresses the expression of Wnt inhibitors such as DKK1/4 and increases the expression of cytoskeletal/extracellular matrix genes.

### Bioactive lipids induce changes in cell morphology and gene expression during hiPSC differentiation

**Figure 3:**
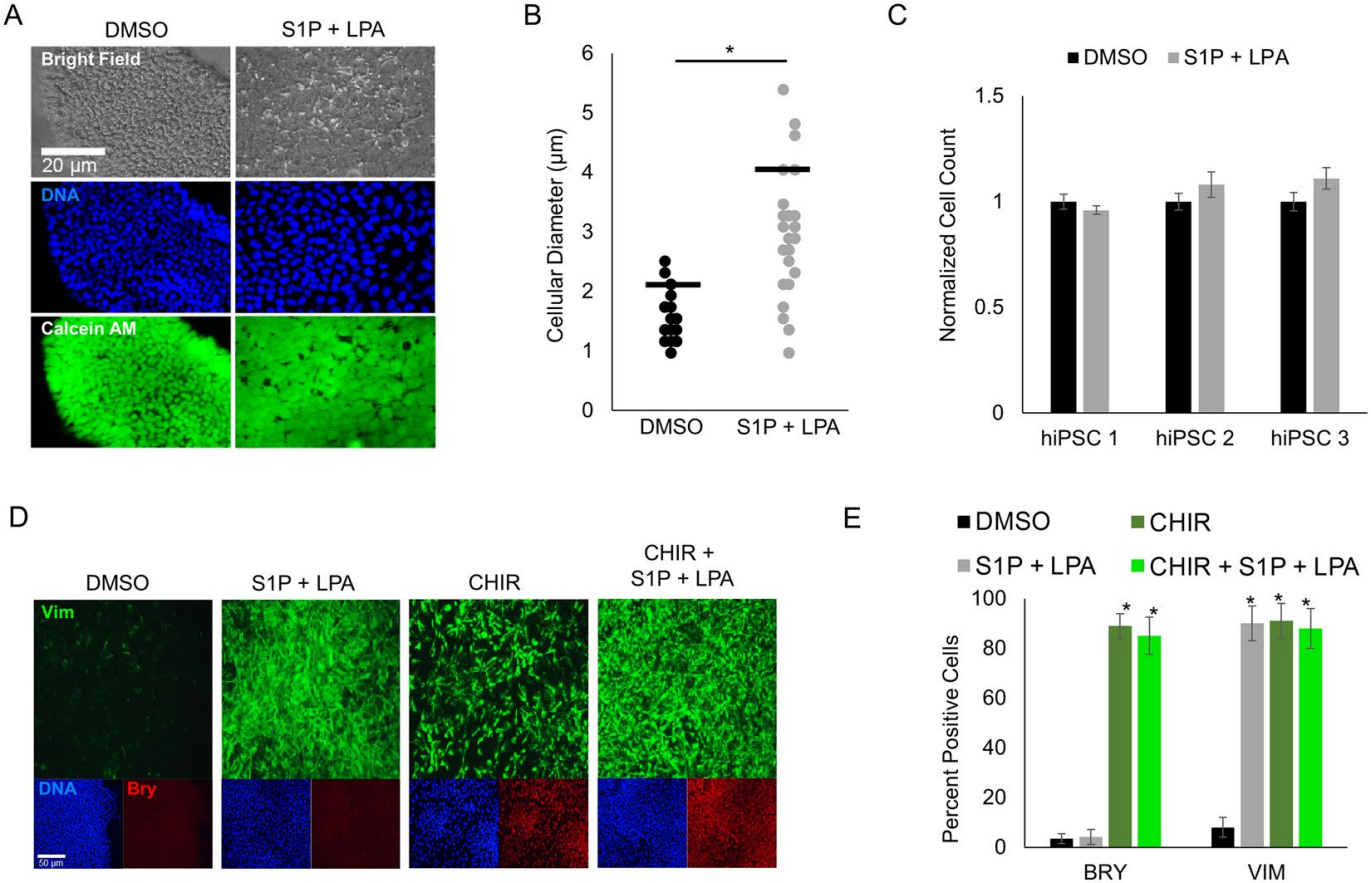
Bioactive lipids S1P/LPA rapidly alter hiPSC morphology and enhance vimentin expression during early cardiac differentiation. (**A**) Immunofluorescence and phase contrast images of hiPSCs treated with S1P and LPA for 24 hours. Calcein AM dye staining membranes the entirety of cell bodies. (**B**) Quantification of cell diameter displayed in μm for hiPSCs treated with DMSO or the combination of S1P and LPA. (**C**) Normalized cell count of 3 separate hiPSC lines following treatment with DMSO or S1P/LPA for 24 hours. N=3 biological replicate experiments. Error bars represent SEM. (**D**) Immunofluorescence staining following 48-hour treatment of hiPSCs with DMSO, GSK3β inhibitor CHIR99021 (CHIR), bioactive lipids S1P + LPA or combination. Intermediate filament protein vimentin (green) marks epithelial-to-mesenchymal transition and Brachyury (red) marks early mesoderm. (**E**) Quantification of vimentin (VIM) and Brachyury T (BRY) positive cells represented as percentages of total cells for control, CHIR, S1P/LPA and combined treatments. Error bars represent standard deviations. Error bars represent standard deviation. Experiment performed in 3 biological replicates. * indicates *P* < 0.05. Cells quantified in N=9 images per condition.

Our microarray data showed an upregulation of the cytoskeletal and extracellular matrix genes (e.g. *COL12A1* and *VIM*) induced by S1P/LPA treatment, suggesting a change in their biological phenotype (Fig. 2E). Furthermore, we noticed a dramatic change in cell morphology and a two-fold increase in cell size within 24 hours of S1P/LPA treatment (Fig. 3A,B). Interestingly, this was accompanied by little to no increase in cell number (Fig. 3C). We validated the changes in gene expression after treatment with bioactive lipids or CHIR from the microarray studies with immunostaining and quantitative PCR analysis (Fig. 3D,E). Interestingly, we found that only CHIR treatment can increase both VIM and Brachyury T (Bry T) expression^25^ while S1P/LPA treatment increased only the expression of VIM without affecting the expression of Bry T (Fig. 3D,E). In conclusion, these results demonstrate a synergistic but mechanistically independent effect of bioactive lipids on Wnt signaling-mediated mesodermal differentiation of hiPSCs to enhance cardiac differentiation.

### Synergistic effects of bioactive lipids and Wnt/β-catenin activation to induce cell cycle activity in terminally-differentiated hiPSC-CMs

Previous studies showed that β-catenin activation is required for ventricular cardiomyocyte proliferation in the mouse fetal heart and in human embryonic stem cell-derived cardiomyocytes, and that metabolic regulation of hiPSC-CM cell cycle arrest can be reversed with activation of β-catenin signaling^26,27^. Given the synergy between bioactive lipids and Wnt/β-catenin signaling to enhance mesodermal differentiation (Figs 2, 3), we examined whether bioactive lipids could also synergize with Wnt/β-catenin signaling to induce proliferation of hiPSC-CMs.

**Figure 4:**
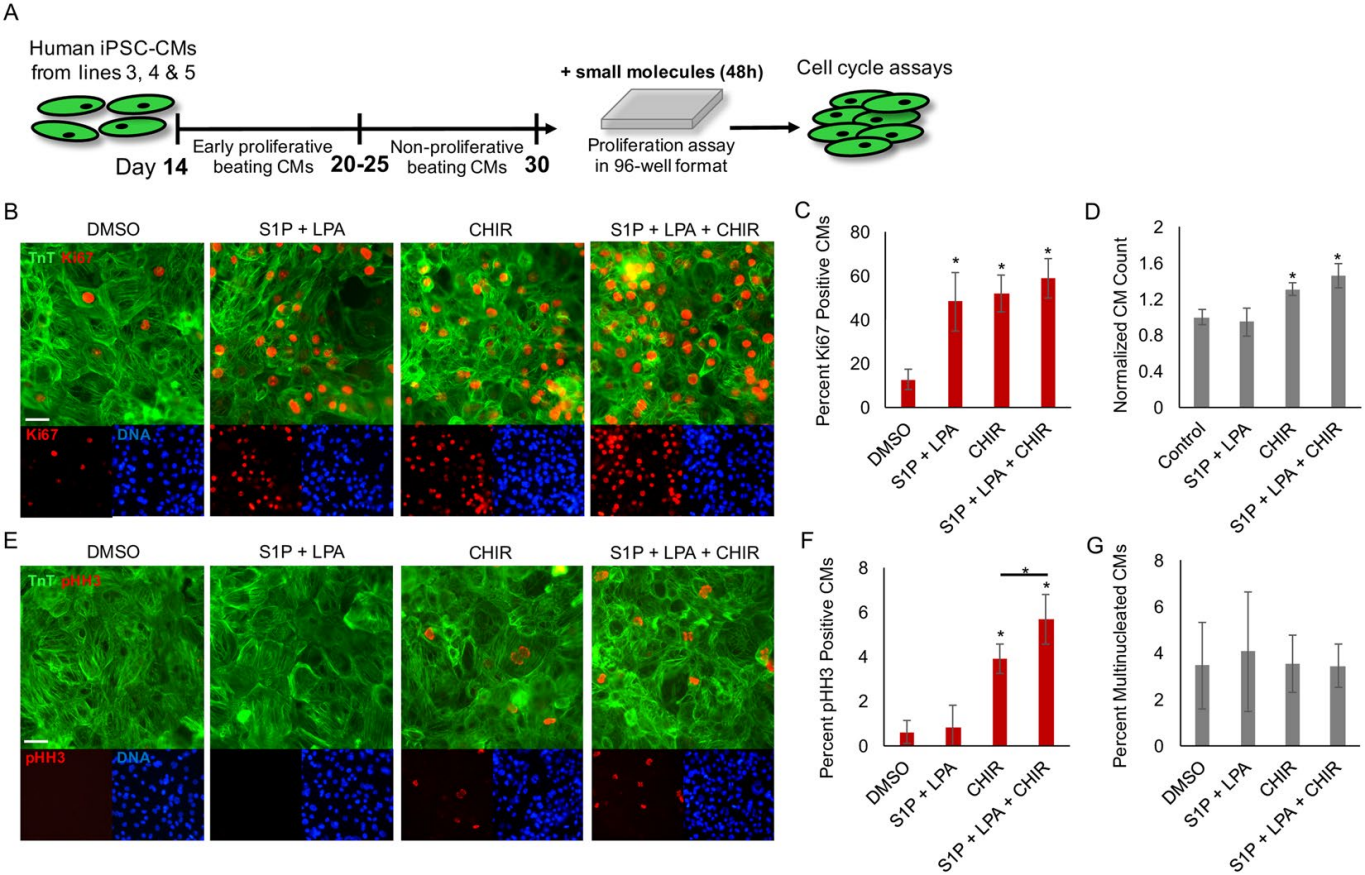
LPA and S1P exhibit cell cycle-inducing effects on hiPSC-CMs. (**A**) Schematic overview of replating hiPSC-CMs at different time-points of differentiation into 96-well format for downstream assays. (**B**) Representative images showing cardiac troponin T (TnT) in green, cell cycle activity marker ki67 in red and nuclear dye (DNA) in blue after 48-hour culture of day 30 hiPSC-CMs with DMSO, S1P/LPA alone, CHIR alone, or CHIR with S1P/LPA. (**C**) Percentage of ki67 positive cardiomyocytes after 48 hours of each treatment. (**D**) Normalized cell count for total number of CMs after 48 hours of treatment for each group. (**E**) Immunofluorescence staining for cardiac troponin T (TnT) in green, mitosis marker phospho Histone H3 (pHH3) in red and nuclear dye (DNA) in blue after 48-hour culture of day 30 hiPSC-CMs with DMSO, S1P/LPA alone, CHIR alone, or CHIR plus S1P/LPA. (**F**) Percentages of mitotic (pHH3) CMs between various treatment groups. (**G**) Percentages of bi- and multinucleated CMs within the indicated treatment groups. *indicates P < 0.05 in comparison to control. N=3 biological replicates. Error bars represent standard deviation.

To address this, we generated well-differentiated hiPSC-CMs (e.g. day 30 or later) using chemically-defined protocols^10,21,28^ (Movie S1) and treated them with S1P/LPA or CHIR or both (Fig. 4A). We first validated the presence of S1P/LPA receptors in these cells (Fig. S2). We then performed immunofluorescence staining for ki67, a marker of cell cycle activity, and phospho Histone H3 (pHH3), a mitosis marker, in cardiac troponin T (TnT) positive cells. We found that treatment with S1P/LPA alone could induce ki67 expression in immature (~day 20–30) as well as well-differentiated (~day 50) hiPSC-CMs to a similar level as treatment with CHIR alone (Fig. 4B,C, S2), a well-known mitogen^29^. However, despite the increase in ki67 expression, S1P/LPA treatment did not lead to an increase in the number of hiPSC-CMs, suggesting that cell (Fig. 4D) and nuclear (Fig. 4E,F) division was incomplete. Nonetheless, treatment with CHIR alone resulted in an increase in cell number and the addition of S1P/LPA to CHIR further enhanced the proliferation of hiPSC-CMs mediated by CHIR, as demonstrated by both the increase in cell number and pHH3 expression (Fig. 4D–F). Two-day treatment with S1P/LPA or CHIR or both did not increase the number of bi- or multinucleated cardiomyocytes (Fig. 4G). Additional functional analyses of day 30 hiPSC-CMs treated with S1P/LPA or CHIR show no significant difference in their beating frequency or contractile force generation when compared with control well-differentiated hiPSC-CMs (Fig. S3). These studies illustrate an additional role for S1P/LPA to regulate cell cycle activity in well-differentiated hiPSC-CMs and synergize with β-catenin signaling to increase hiPSC-CM proliferation.

### Bioactive lipids activate ERK signaling in hiPSC-CMs

**Figure 5:**
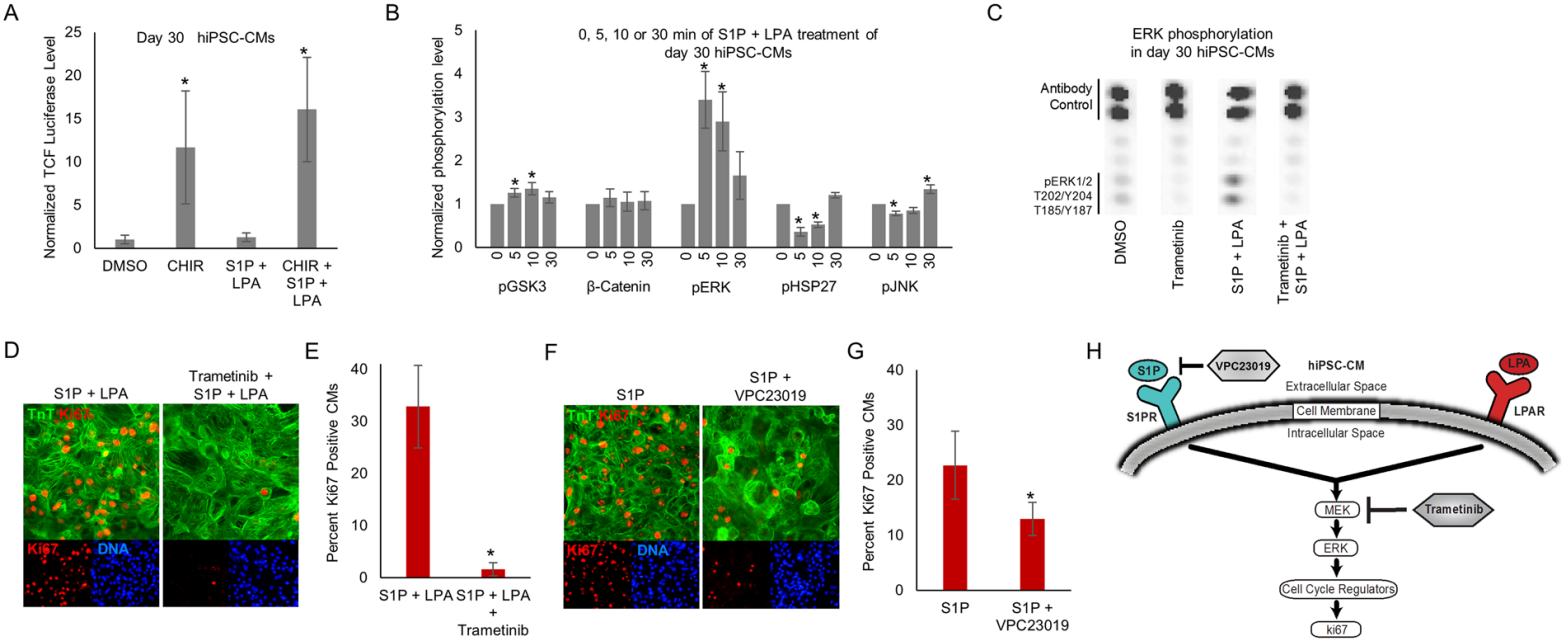
Reactivation of cell cycle in hiPSC-CMs with S1P and LPA is dependent on ERK signaling. (**A**) Luciferase luminescence intensity after transfection of day 30 hiPSC-CMs with TOPFlash Wnt signaling pathway activity reporter and 2-hour treatment with CHIR, S1P/LPA or both. The data shown represents fold increase over DMSO control. (**B**) Quantification of kinase assays illustrating alterations in hiPSC-CM kinome phosphorylation in response to 0, 5, 10, and 30-minute S1P/LPA treatment. Data expressed as means ± SEM. *indicates P < 0.05. (**C**) Representative kinase assay conducted in day 30 hiPSC-CMs treated with and without small molecule MEK inhibitor trametinib, with and without S1P/LPA (10 μM each). Spots corresponding to ERK phosphorylation and antibody control are labeled. (**D**) Immunofluorescence for cardiac troponin T (TnT) (green), ki67 (red) and nuclear DNA (blue) in day 30 hiPSC-CMs treated with S1P and LPA in the presence or absence of MEK inhibitor trametinib. (**E**) Quantification of the percentages of ki67 positive cardiomyocytes (CMs) in (**D**). *indicates P < 0.001. (**F**) Immunofluorescence for cardiac troponin T (TnT) (green), ki67 (red) and nuclear DNA (blue) in day 30 hiPSC-CMs treated with bioactive lipid S1P in the presence or absence of 5 μM S1P receptor antagonist VPC23019. (**G**) Quantification of the percentages of ki67 positive CMs after S1P treatments with or without 5 μM VPC23019. (**H**) Model illustrating the link between bioactive lipids and the canonical MAPK/MEK/ERK signaling pathway in differentiated hiPSC-CMs. N=4.

To determine the mechanism through which S1P/LPA regulate cell cycle activity in hiPSC-CMs, we first evaluated whether S1P and LPA treatment can directly stimulate Wnt/β-catenin signaling in day 30 cardiomyocytes using the TOPFlash (TCF/LEF – luciferase) reporter. We found that while the canonical Wnt signaling, as measured by the TOPFlash reporter, was activated in the presence of CHIR alone, the treatment with S1P/LPA was unable to increase TCF/LEF-luciferase activity (Fig. 5A), suggesting the effect of bioactive lipids is not mediated directly by Wnt signaling in hiPSC-CMs. Given the well-known role of Hippo/Yap signaling in regulating cardiomyocyte and non-myocyte proliferation and regeneration^30-34^, we assessed the ability of bioactive lipids to promote nuclear accumulation of Yap (Fig. S4). Surprisingly, we found a strong nuclear YAP localization (>85–90% of all nuclei counted) at baseline in hiPSC-CMs and many other cell types including undifferentiated hiPSCs and hiPSC-derived non-myocytes (Fig. S4A). Consequently, we saw no increase in nuclear YAP accumulation following bioactive lipid treatment (Fig. S4B).

To further elucidate the mechanism through which S1P/LPA regulate cell cycle activity in hiPSC-CMs, we performed a broad kinase phosphorylation panel screening in day 30 hiPSC-CMs after treatment with S1P/LPA (Table S1). Notably, we observed a rapid upregulation in phosphorylation of ERK1/2, a known regulator of cell cycle activity, after S1P/LPA treatment (Fig. 5B)^35^. We also observed a down-regulation in phosphorylation of HSP27 within 5 minutes of treatment with S1P/LPA and subtle alterations in GSK3β and JNK phosphorylation (Fig. 5B). Consistent with the lack of a direct effect of S1P/LPA on canonical Wnt/β-catenin signaling, the level of β-catenin remained unchanged following treatment with S1P/LPA. Analysis of S1P/LPA treatment in hiPSCs undergoing cardiac differentiation revealed that ERK1/2 phosphorylation was not active in day 0 undifferentiated hiPSCs (Fig. S5) despite the effect of S1P/LPA on CHIR-induced mesodermal induction (Fig. 2).

To address whether S1P/LPA promotes ERK1/2 phosphorylation in hiPSC-CMs through stimulation of the MAPK/MEK/ERK signaling cascade, we treated hiPSC-CMs with S1P/LPA in the presence or absence of trametinib, a small molecule inhibitor of MEK signaling upstream of ERK^36^. We found that the ability of S1P/LPA to activate ERK phosphorylation in hiPSC-CMs was abolished in the presence of trametinib, confirming that S1P/LPA-induced ERK phosphorylation is mediated by MAPK/MEK/ERK signaling (Figs 5C, S6). Next, we examined the requirement of MAPK/MEK/ERK signaling on S1P/LPA-induced cell cycle activity by treating hiPSC-CMs with S1P/LPA in the presence and absence of trametinib and assessed the expression of ki67 in hiPSC-CMs (Fig. 5D,E). We found that trametinib treatment abolished the S1P/LPA-induced upregulation of ki67. This confirms the role of MAP/MEK/ERK signaling in S1P/LPA-mediated cell cycle activation in hiPSC-CMs.

To assess whether S1P receptor signaling is involved S1P-mediated hiPSC-CM cell cycle activation, we cultured hiPSC-CMs with S1P alone or together with its receptor antagonist, VPC23019^37^ and found that the expression of ki67 in S1P-treated hiPSC-CMs was reduced in the presence of VPC23019 (Fig. 5F,G). This supports a role for the S1P receptor to mediate bioactive lipid-induced hiPSC-CM cell cycle activation (Fig. 5H). Finally, although we observed an increase in cell cycle activity in hiPSC-CMs in response to S1P/LPA treatment, we did not observe a shift in maturation status or subtype identity of hiPSC-CMs after S1P/LPA treatment (Fig. S7).

## Discussion

Bioactive lipids have garnered significant attention for their ability to influence cell proliferation, differentiation, and migration in a variety of non-cardiomyocyte cell types^38^. In particular, they have shown an ability to modulate Wnt/β-catenin and Hippo signaling pathways, both of which are critical for influencing cardiomyocyte proliferation, development, and differentiation^7,22,26,30–32^. In this study, we employed an hiPSC model of cardiogenesis to examine the effects of bioactive lipids on hiPSC cardiac differentiation and cardiomyocyte generation. Interestingly, we found highly stage-specific roles for S1P/LPA during hiPSC-CM differentiation. When administered to undifferentiated hiPSCs, either alone or in combination with CHIR, S1P/LPA increased nuclear β-catenin level and enhanced mesodermal induction. After the completion of cardiomyocyte differentiation, the addition of S1P/LPA initiated cell cycle re-entry in hiPSC-CM by activating MAPK/MEK/ERK signaling and enhanced CHIR-induced cardiomyocyte proliferation. These findings illustrate the versatility of the hiPSC differentiation platform for studying the effects of signaling pathways on human cardiomyocyte development. In addition, the ability to mass-produce differentiated human cardiomyocytes by bioactive lipid treatment will facilitate the development of high throughput assays for cardiac disease modeling and discovery of new molecules for future regenerative applications.

The ability of S1P and LPA to rapidly induce morphological and gene expression changes in undifferentiated hiPSCs was quite striking and a somewhat unexpected finding in our study. We observed a rapid change in hiPSC morphology within 12–24 hours from the initiation of S1P/LPA treatment. These changes were also accompanied by an increase in the expression of the intermediate filament protein vimentin in hiPSCs, a finding that suggests the induction of epithelial-to-mesenchymal transition (EMT). However, S1P/LPA treatment did not lead to a decreased expression of Nanog at 24 hrs after treatment, suggesting a more mesodermal specific, rather than global, effect of S1P/LPA on hiPSC cardiac differentiation (Fig. S1B). A hallmark of EMT is the loss of cell-cell contact normally mediated by adherens junction complexes^39^. Lysophospholipids are well-established for their ability to dissociate these adherens junctions, dramatically loosen cell-cell contact, and release adherens junction-bound β-catenin into the cytoplasm^22,40^. Importantly, β-catenin also functions as a downstream nuclear transcriptional effector for activating Wnt signaling^7^. Indeed, we observed that treatment with S1P/LPA rapidly induces β-catenin cytoplasmic and nuclear accumulation in hiPSCs. Thus, S1P/LPA treatment synergizes with CHIR-mediated GSK3β inhibition to enhance the overall pool of cytoplasmic β-catenin and promotes its nuclear entry (Fig. 2D). Beyond promoting an increase in the cytoplasmic pool of β-catenin, S1P/LPA treatment appears to induce an increase in vimentin expression via a different mechanism, since the presence of Wnt inhibitor fails to abrogate the ability of S1P/LPA to simulate vimentin expression (data not shown). Further work will be necessary to identify additional downstream signaling events responsible for the biological effects of S1P/LPA on vimentin expression.

While the increase in β-catenin nuclear localization could be due to stabilization of β-catenin (i.e. prevention of GSK3β-mediated degradation) or greater release of β–catenin from E-cadherin at the plasma membrane, addressing the relative contribution of these mechanisms of β-catenin increase in the nucleus is beyond the scope of the current study. However, the inability of S1P/LPA to directly induce early mesoderm markers such as Bry T (Fig. 3D,E) supports their independent effects on hiPSC differentiation besides facilitating β-catenin nuclear translocation. This was further supported by the absence of a strong effect of S1P/LPA on LEF/TCF reporter expression (Figs 2C, 5A) suggesting the involvement of Wnt/β-catenin independent mechanisms on hiPSCs cardiomyocyte differentiation. Identification of additional signaling pathways involved in mesodermal induction by S1P/LPA may help to further improve hiPSC cardiac differentiation.

Our finding that S1P/LPA treatment induced a strong and rapid up-regulation of MAPK/MEK/ERK signaling in well-differentiated hiPSC-CMs was quite intriguing. The ability of S1P/LPA to induce ERK signaling, a known regulator of cell proliferation, has been described in other cell types^38^. We confirmed that the MAPK/MEK/ERK pathway is required for S1P/LPA-induced ERK phosphorylation and cell cycle reentry by showing that treatment with trametinib, a MEK inhibitor, effectively abolished these effects (Fig. 5D,E). The involvement of MEK signaling is further supported by the S1P/LPA-induced down-regulation of HSP27 phosphorylation, a previously reported target of MEK that opposes ERK phosphorylation^41^. Interestingly, the PI3-Akt pathway, a known pathway involved in cardiomyocyte proliferation^42^, was not activated at baseline or by S1P/LPA treatment. This may be because these hiPSC-CMs are phenotypically immature or lack an optimal culture condition for stimulating PI3K-Akt signaling. Our findings are also consistent with a recent study demonstrating involvement of ERK and YAP signaling in adult cardiomyocyte division^43^ and suggest that *in vivo* delivery of S1P/LPA may also enhance cardiomyocyte division. However, further studies will be necessary to validate this hypothesis. Given the relative immaturity of hiPSC-CMs, our results here may only apply to fetal or neonatal cardiomyocytes *in vivo*. Additionally, bioactive lipids other than S1P and LPA should also be tested at multiple concentrations to see what effect they would have on mesodermal expansion and/or cardiomyocyte proliferation.

## Conclusion

Our study addressed, for the first time, the stage specific effects of bioactive lipids on hiPSC differentiation and hiPSC-CM proliferation and demonstrated a role for bioactive lipids to enhance human iPSC differentiation into cardiomyocytes. While the efficiency of hiPSC differentiation into CMs has increased remarkably in recent years, there remain significant variations among human iPSC lines and between different differentiation batches from the same line. Thus, identifying the ability of new signaling molecules to further augment CM differentiation of human iPSCs is of significant interest to the cardiovascular and stem cell biology community. In summary, our study provides a greater understanding of the role of bioactive lipids in cardiovascular biology and a novel means of enhancing the production of hiPSC-CMs that can be used for downstream applications in cardiovascular disease modeling, drug screening, and regenerative medicine.

## Experimental Procedures

An expanded Experimental Procedures section is available in the Supplemental Materials.

### Chemically-defined differentiation of hiPSC-CMs

To produce human cardiomyocytes from pluripotent stem cells, hiPSCs were differentiated into hiPSC-CMs with a chemically-defined cardiomyocyte differentiation protocol. These hiPSC-CMs were maintained in RPMI 1640 media supplemented with recombinant human albumin and ascorbic acid (CDM3)^10^. Briefly, hiPSCs were first treated with a small molecule inhibitor of GSK3β signaling, CHIR99021, to activate the Wnt signaling pathway. Two days later, cells were treated with a Wnt signaling inhibitor, Wnt-C59, until day 4. Afterwards, CDM3 media without any small molecules was changed every two days. To purify cardiomyocytes, the cell population was glucose-starved and supplemented with 5 mM sodium DL-lactate for 2 to 4 days to metabolically select hiPSC-CMs^44^. When replating hiPSC-CMs, cells were dissociated with TrypLE Express (Life Technologies) and reseeded on Matrigel-coated plates.

### 96-well differentiation, imaging, and quantitative viability assays

For 96-well hiPSC-CM differentiation assays, hiPSCs were plated in Matrigel-coated 96-well plates at 1000 cells per well and allowed to adhere for 4 days. The hiPSCs were subsequently treated with bioactive lipids at the indicated concentrations and durations and assessed following day 8 of the chemically-defined hiPSC-CM differentiation protocol. Immunostaining using previously-published protocols was conducted to qualitatively assess cell viability and cardiomyocyte differentiation efficiency^45^. Fluorescence intensity and cell number was quantified using ImageJ software. For quantitative viability measurements, cells were treated with CellTiter-Glo 2.0 Viability Assay (Promega) or PrestoBlue reagent (Life Technologies) per manufacturer-recommended procedures. 96-well imaging and viability assays were conducted using a Cytation 5 plate reader/imager (BioTek Instruments). Prism (GraphPad) was utilized for graph generation and statistical analysis. Confocal imaging was performed using a Zeiss LSM 510Meta microscope (Carl Zeiss) using Zen software.

### Small molecules

S1P and LPA were obtained from Sigma Aldrich and dissolved in water at 1 mM and 10 mM stock solutions. S1P and LPA were applied in 10 μM final concentrations unless otherwise specified. C59 and CHIR99021 were obtained from Tocris Bioscience and dissolved in DMSO at 10 mM stock concentrations. S1P antagonist VPC 23019 was obtained from Tocris Bioscience and dissolved in acidified DMSO.

### Kinase phosphorylation profiling

Phosphorylation of human kinases and other phosphoproteins (Table S1) was determined using a Human Phospho-Receptor Tyrosine Kinase (RTK) Array or Human Phospho-Kinase Antibody Array (R&D Systems). Cells were treated with bioactive lipids at indicated concentrations and durations. An RTK or phospho-kinase panel was incubated overnight with 10 mg cell protein lysate and subsequently with an anti-phospho-tyrosine-horseradish peroxidase antibody to assess phosphorylation. Blots were developed using a Gel Doc XR (BioRad). Phosphorylation intensity was determined using ImageJ software.

### Luciferase luminescence measurements

HiPSCs and day 30 hiPSC-CMs were replated in 96-well plates and cultured for 2–3 days before transfection with Lipofectamine (Invitrogen) and TOPFlash (TCF/LEF) luciferase Wnt signaling reporter plasmid (M50, Addgene) at 100 ng/well. After 48 hours, media was changed, and cells were subjected to different treatments for 2 hours before lysis and luciferase (Promega) luminescence was measured with a standard luminescence plate reader.

### Gene expression

Quantitative real-time PCR was used to assess the gene expression level of specific gene of interest following bioactive lipids treatment. RNA was isolated using an RNeasy Plus kit (QIAGEN), and cDNA was produced using the High-Capacity RNA-to-cDNA kit (Applied Biosystems). Real-time PCR was performed with CFXTM Connect Real-Time System (BIO-RAD) using the USB® HotStart-IT® SYBR® Green qPCR Master Mix (2×) (Affymetrix). qPCR reactions were performed in duplicate, normalized to the reference gene GAPDH, and assessed using the comparative Ct method^46^. For more comprehensive transcriptome analysis of hiPSCs following bioactive lipids treatment, a GeneChip® Human Gene 1.0 ST DNA Microarray was used (Affymetrix).

### Statistical Methods

Data presented as mean ± standard deviation unless otherwise specified. Comparisons were conducted via Student’s t-test with significant differences (*) defined by *P* < 0.05, unless otherwise specified. For microarray, multiple *P*-value comparisons were made using a one-way between-subject ANOVA (*P* < 0.05) using Affymetrix Transcriptome Analysis Console 2.0 software.

### eTOC Blurb

Wu and colleagues demonstrate a stage-specific role of bioactive lipids to enhance human hiPSC cardiac differentiation (early) and cardiomyocyte cell cycle activation (late). These findings will help improve hiPSC-CM generation for cardiac disease modeling, precision medicine, and regenerative therapies.

## Electronic supplementary material

Supplemental Movie 1

Supplementary Information
