# Supplementary material for: Stage-specific Effects of Bioactive Lipids on Human iPSC Cardiac Differentiation and Cardiomyocyte Proliferation
Source: Sci Rep. 2018 Apr 26;8:6618. doi: 10.1038/s41598-018-24954-3 (PMC5920079; doi:10.1038/s41598-018-24954-3)
Supplement: Supplementary file 2 — Supplementary Information [file 41598_2018_24954_MOESM2_ESM.pdf]

SUPPLEMENTAL MATERIALS

Stage-specific Effects of Bioactive Lipids on Human iPSC Cardiac Differentiation and Cardiomyocyte Proliferation

Arun Sharma[1, 2±*], Yuan Zhang[1*], Jan W. Buikema[1, 3 *], Vahid Serpooshan[1], Orlando Chirikian[1], Nina Kosaric[3], Jared M. Churko[1], Elda Dzilic[1, 4], Alice Shieh[1], Paul W. Burridge[1+], Joseph C. Wu[1, 3, 5], Sean M. Wu[1, 3, 5]

*****These authors contributed equally to this work.

Affiliations:
[1]Stanford Cardiovascular Institute, Stanford University School of Medicine, Stanford CA, 94305, USA
[2]Institute for Stem Cell Biology and Regenerative Medicine, Stanford University School of Medicine, Stanford CA, 94305, USA
[3]Department of Cardiology, University Medical Center Utrecht, Utrecht University, 3508 GA Utrecht, The Netherlands
[4]Department of Cardiovascular Surgery, German Heart Centre Munich, Technical University Munich, Munich, Germany
[5]Department of Medicine, Division of Cardiology, Stanford University School of Medicine, Stanford CA, 94305, USA

Keywords: cell signaling, cardiomyocytes, cardiomyocyte proliferation, cardiac differentiation, induced pluripotent stem cells, cell cycle, differentiation, proliferation

[±]Current address: Harvard Medical School, New Research Building Room 256, 77 Avenue Louis Pasteur, Boston, MA 02115, USA
[+]Current address: Department of Pharmacology and Center for Pharmacogenomics, Northwestern University Feinberg School of Medicine, USA

Contact:
Sean M. Wu, MD, PhD
Lokey Stem Cell Building, Rm G1120A
265 Campus Drive
Stanford, CA 94305-5454
Email: smwu@stanford.edu
Phone: 650-724-4498
Fax: 650-724-4689

This file contains:

SUPPLEMENTAL FIGURES

Figure S1: Bioactive lipids S1P and LPA increase nuclear beta-catenin but do not induce early mesodermal differentiation.

Figure S2: hiPSC-CMs express relevant S1P and LPA receptors upon terminal differentiation and respond to S1P/LPA treatment.

Figure S3: Motion-derived contractility parameters in day 30 hiPSC-CMs.

Figure S4: LPA is unable to enhance YAP nuclear accumulation in hiPSC-CMs.

Figure S5: Bioactive lipids do not change ERK phosphorylation in undifferentiated hiPSC.

Figure S6: S1P and LPA activate MAPK/MEK/ERK signaling in hiPSC-CMs.

Figure S7: S1P and LPA do not alter maturation or subtype specification in hiPSC-CMs.

SUPPLEMENTAL TABLES

Table S1: Panel of different phosphorylated kinases as screened in hiPSCs and hiPSC-CMs treated with bioactive lipids S1P and LPA.

SUPPLEMENTAL MOVIES

Movie S1: hiPSC-CMs after purification via glucose deprivation.
Supplemental Experimental Procedures.

SUPPLEMENTAL FIGURES

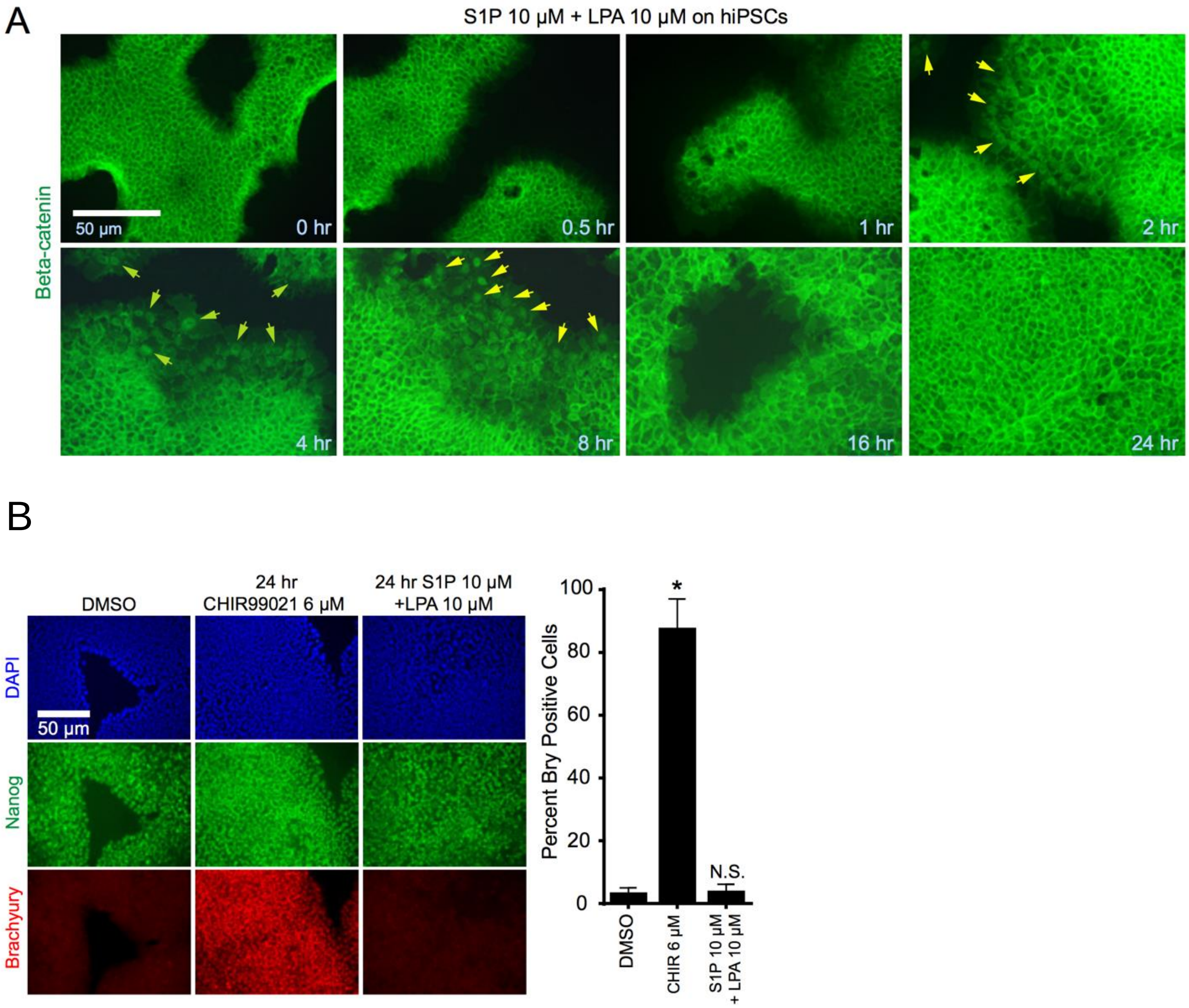

Figure S1: Bioactive lipids S1P and LPA increase nuclear beta-catenin but do not induce early mesodermal differentiation. A) Time course study on nuclear beta-catenin accumulation after 0, 0.5, 1, 2, 4, 8, 16 and 24 hour (hr). Arrows indicate cells expressing profound nuclear beta-catenin. **B**) Immunofluorescence for pluripotency marker Nanog (green), early mesoderm marker Brachyury (red) and DAPI (blue) in hiPSCs cultured for 24 hr with GSK3 inhibitor CHIR99021, bioactive lipids S1P and LPA or DMSO control. Graph represents quantification of percentages Brachyury (Bry) positive cells for the listed treatments. Error bars represent SEM. *indicates P<0.05. N.S. = not significant. N=3.

A

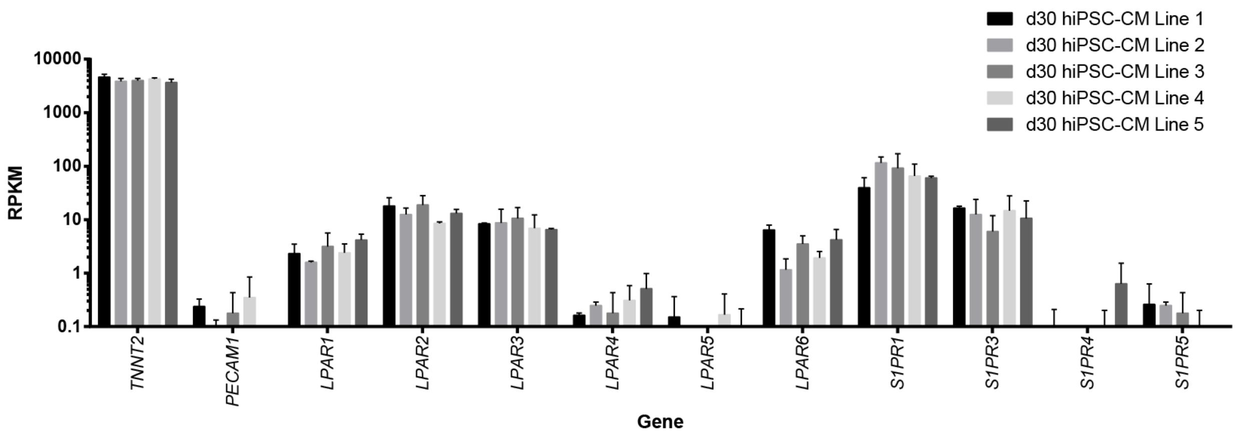

B

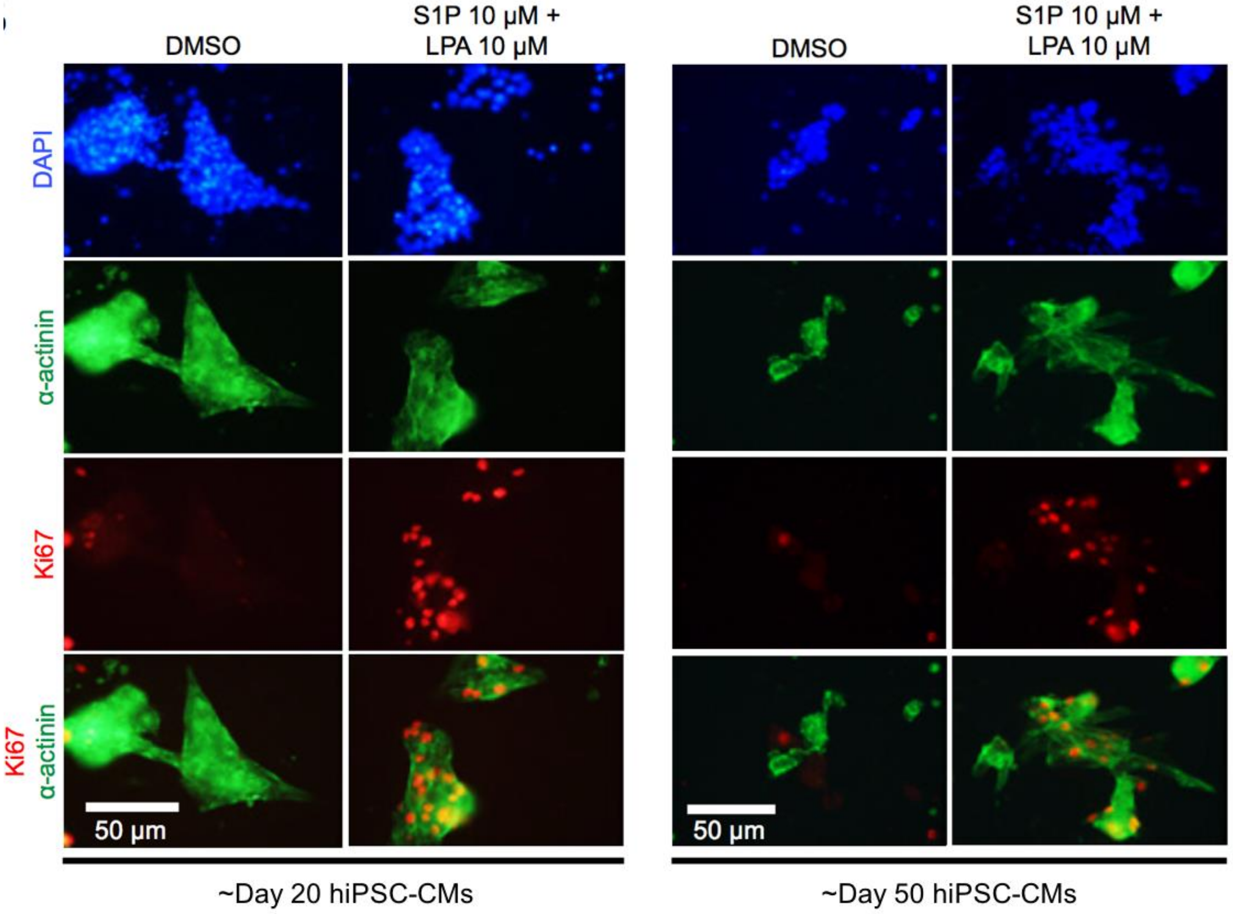

Figure S2: hiPSC-CMs express relevant S1P and LPA receptors upon terminal differentiation and respond to S1P/LPA treatment. A) Transcriptome profiling of day 30 hiPSC-CMs from 5 different hiPSC-CM lines. High *TNNT2* and low *PECAM1* indicates high cardiomyocyte purity and low endothelial cell contamination, respectively. Expression profiling conducted using IonTorrent Ampliseq transcriptome profile. Error bars represent SEM. **B**) Bioactive lipids LPA and S1P induce ki67 expression at different time points of terminal differentiation. Immunofluorescence for alpha-actinin (green), ki67 (red) and DAPI (blue) after 48-hour treatment with S1P and LPA or DMSO in day ~20 and ~50 hiPSC-CMs.

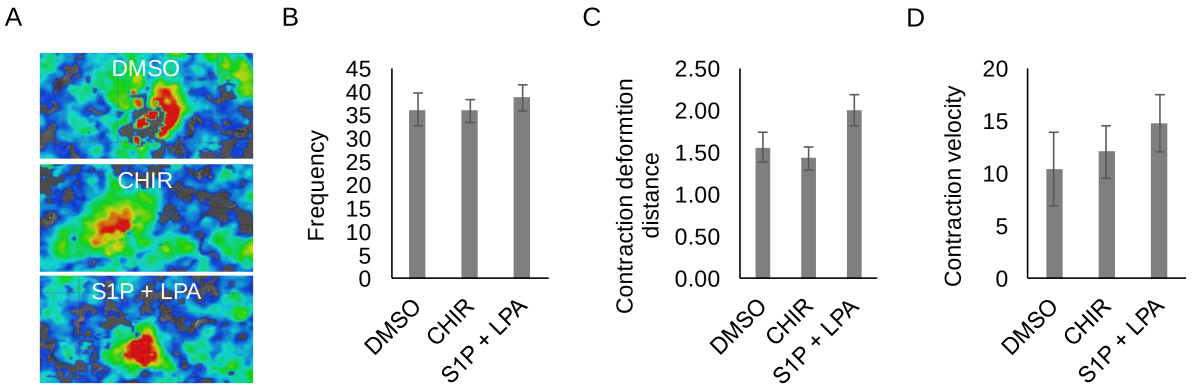

Figure S3: Motion-derived contractility parameters in day 30 hiPSC-CMs. A) Representative heatmaps of day 30 hiPSC-CMs treated with DMSO, CHIR99021 or S1P/LPA generated from high resolution and frequency movies, red = high motion, blue = low motion. Graphs displaying contraction frequency (beats/minute) **B**), contraction deformation distance **C**), and contraction velocity **D**). Data represented as means. Error bars indicate SEM.

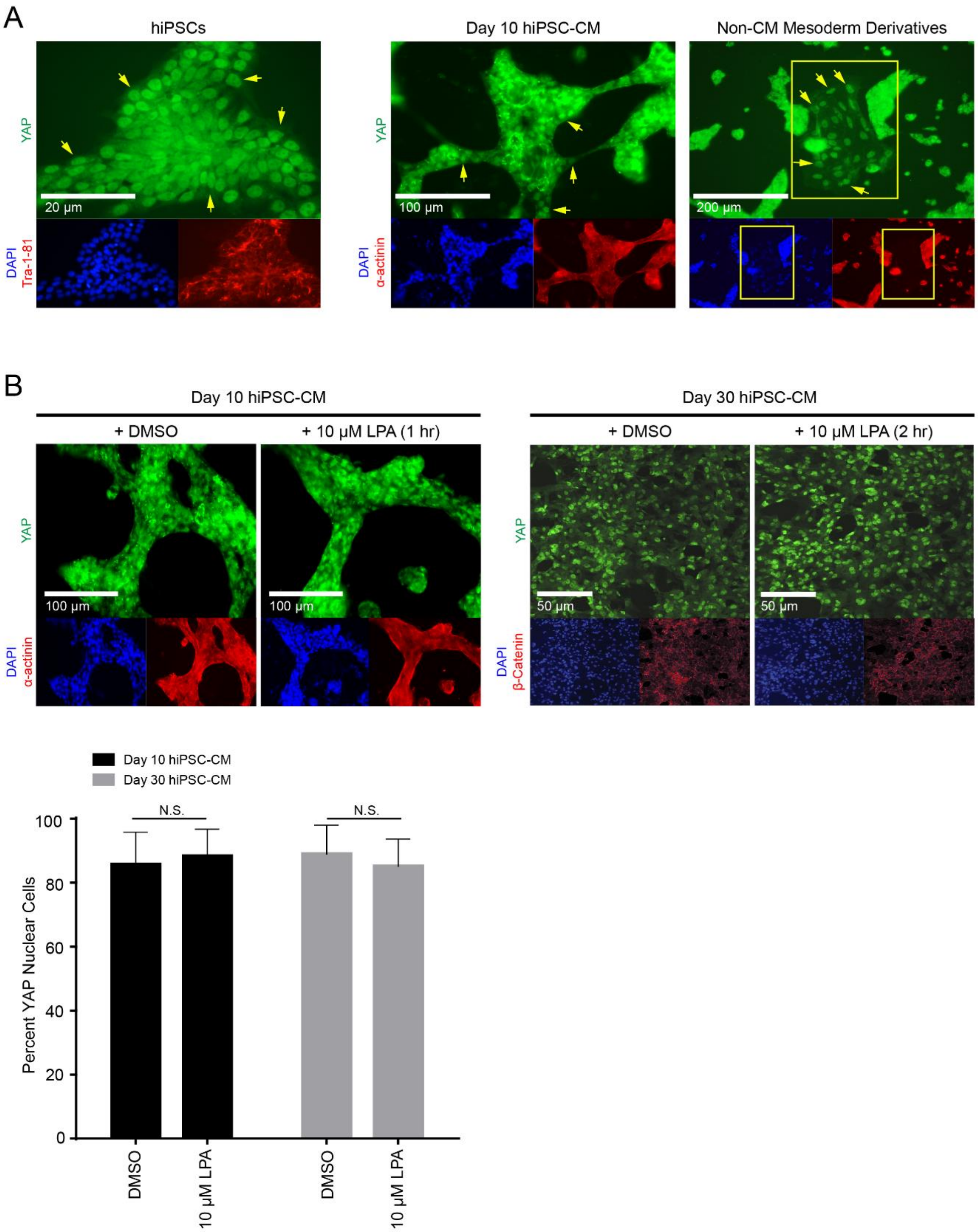

Figure S4: LPA is unable to enhance YAP nuclear accumulation in hiPSC-CMs. A) Immunofluorescence staining in untreated hiPSCs, hiPSC-CMs, and non-CM mesodermal derivatives (indicated by yellow boxes) for DAPI (blue), total YAP (green), and cell-type specific markers (red): Tra-1-81 for hiPSCs and alpha-actinin for hiPSC-CMs. YAP is localized to the nuclei in all three cell types at baseline, as indicated by representative cells marked by yellow arrows. **B)** YAP immunofluorescence for purified day 10 or day 30 hiPSC-CMs with and without treatment with LPA, stained for downstream Hippo pathway transcriptional effector YAP (green). No significant (N.S.) increase in YAP nuclear accumulation or β-catenin translocation seen after LPA treatment on day 10 or day 30 hiPSC-CMs. Cells quantified in N=9 images per condition. Data are expressed as means ± STD.

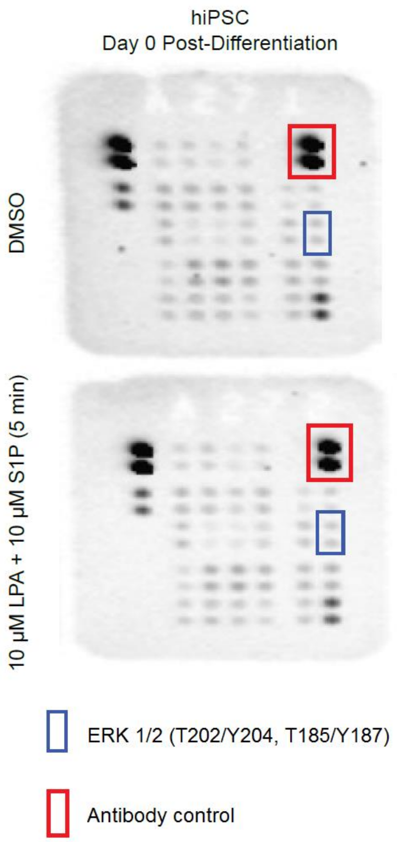

Figure S5: Bioactive lipids do not change ERK phosphorylation in undifferentiated hiPSCs. ERK1/2 phosphorylation conducted in undifferentiated hiPSC with and without 5 minute S1P and LPA (S+L) treatment. Spots corresponding to ERK1/2 phosphorylation and antibody control are labeled.

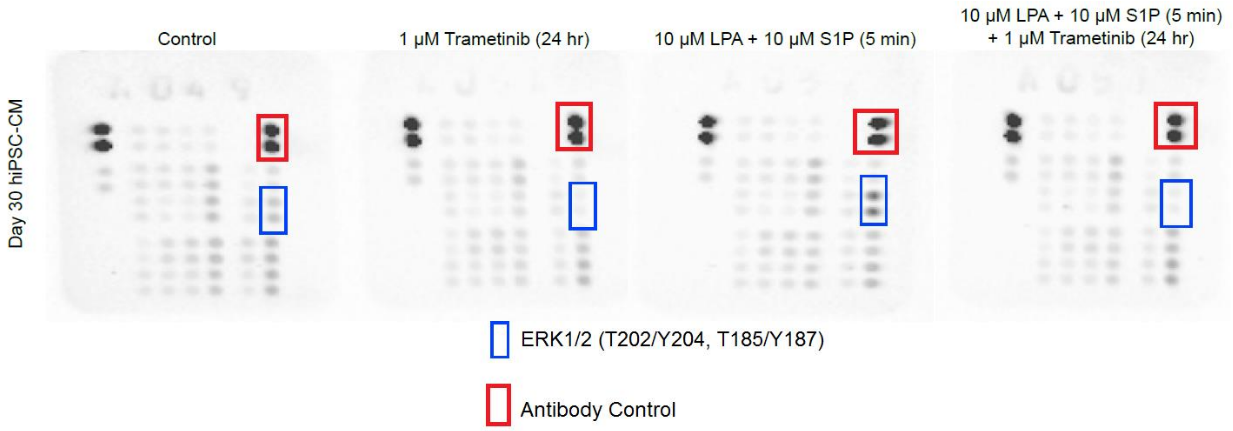

Figure S6: S1P and LPA activate MAPK/MEK/ERK signaling in hiPSC-CMs. Full version of Figure 5C. Kinase assay conducted in day 30 hiPSC-CMs treated with and without small molecule MEK inhibitor trametinib, with and without S1P/LPA (10 µM each). Spots corresponding to ERK phosphorylation and antibody control are labeled.

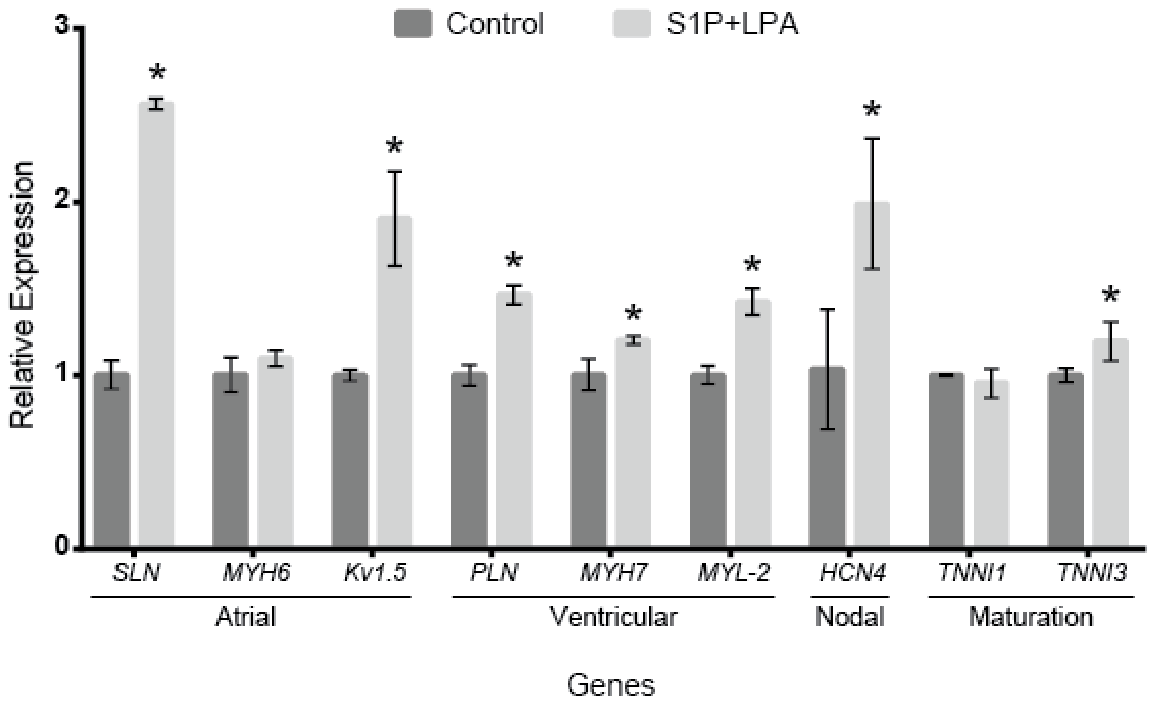

Figure S7: S1P and LPA do not alter maturation or subtype specification in hiPSC-CMs. QPCR gene expression analysis conducted in day 30 hiPSC-CMs treated with and without S1P/LPA (10 µM each). Genes corresponding to atrial, ventricular, and nodal subtypes, as well as maturation markers, are labeled. * indicates $p<0.05$.

SUPPLEMENTAL TABLES

**Protein (Residue)**	**Code**
Secondary Antibody Control	A1/A2
p38a (T180/Y182)	A3/A4
*ERK1/2 (T202/Y204, T185/Y187)	A5/A6
*JNK 1/2/3 (T183/Y185, T221/Y223)	A7/A8
*GSK-3a/ß (S21/S9)	A9/A10
Blank	B1/B2
EGFR (Y1086)	B3/B4
MSK1/2 (S376/S360)	B5/B6
AMPKα1 (T183)	B7/B8
Akt 1/2/3 (S473)	B9/B10
TOR (S2448)	C1/C2
CREB (S133)	C3/C4
*HSP27 (S78/S82)	C5/C6
AMPKα2 (T172)	C7/C8
Total ß-Catenin	C9/C10
Src (Y419)	D1/D2
Lyn (Y397)	D3/D4
Lck (Y394)	D5/D6
STAT2 (Y689)	D7/D8
STAT5a (Y694)	D9/D10
Fyn (Y420)	E1/E2
Yes (Y426)	E3/E4
Fgr (Y412)	E5/E6
STAT6 (Y641)	E7/E8
STAT5b (Y699)	E9/E10
Hck (Y411)	F1/F2
Chk-2 (T68)	F3/F4
FAK (Y397)	F5/F6
PDGFRß (Y751)	F7/F8
STAT5a/b (Y694/Y699)	F9/F10
Secondary Antibody Control	G1/G2
PRAS40 (T246)	G3/G4
Blank	G5/G6
Blank	G7/G8
PBS (Negative Control)	G9/G10

Supplemental Table S1: Panel of different phosphorylated kinases as screened in hiPSCs and hiPSC-CMs treated with bioactive lipids S1P and LPA. In table, proteins with an asterisk (*) indicate significant alterations (P<0.05) in phosphorylation following S1P/LPA treatment (see also Figure 5).

SUPPLEMENTAL MOVIES

Movie S1. hiPSC-CMs after purification via glucose deprivation. Following differentiation, hiPSC-CMs begin to spontaneously contract at approximately day 8-10 after cardiac differentiation is initiated. Following glucose deprivation, cell sheets contained a purer population of hiPSC-CMs. Movie at 10x magnification.

SUPPLEMENTAL EXPERIMENTAL PROCEDURES

Derivation of human induced pluripotent stem cells (hiPSCs). All the protocols for this study were approved by the Stanford University Institutional Review Board. Reprogramming was conducted according to a previously-published protocol (Churko et al 2013). In summary, peripheral blood mononuclear cells (PBMCs) were obtained by conducting a standard blood draw from consenting individuals and isolated using a Ficoll gradient separation. PBMCs were reprogrammed using a Sendai virus vector expressing *OCT4*, *KLF4*, *SOX2*, and *MYC* (OKSM) (Life Technologies) following the protocol supplied by the manufacturer. Approximately one month after reprogramming, hiPSC clones were isolated and cultivated on growth factor-reduced Matrigel (Corning)-coated 6-well tissue culture dishes (Greiner) in E8 pluripotent stem cell culture medium (Life Technologies).

Gene expression. Expression of bioactive lipid receptors in hiPSC-CMs was determined using Ion AmpliSeq (Life Technologies). RNA was extracted with the RNeasy Micro kit (Qiagen). cDNA libraries were synthesized using the Ion AmpliSeq Transcriptome Human Gene Expression kit. Libraries were added to Ion PI chips and added to the Ion Chef instrument for template preparation. Transcriptome sequencing was conducted on an Ion Proton sequencing system (Life Technologies). For expression analysis of hiPSC-CMs following lipid treatment, a GeneChip® Human Gene 1.0 ST DNA Microarray was used (Affymetrix), or qPCR expression analysis was conducted (BioRad).
